# Evolution of Chromosomal *Clostridium botulinum* Type E Neurotoxin Gene Clusters: Evidence Provided by Their Rare Plasmid-Borne Counterparts

**DOI:** 10.1093/gbe/evw017

**Published:** 2016-02-14

**Authors:** Andrew T. Carter, John W. Austin, Kelly A. Weedmark, Michael W. Peck

**Affiliations:** ^1^Gut Health and Food Safety, Institute of Food Research, Norwich Research Park, Norwich, United Kingdom; ^2^Bureau of Microbial Hazards, Health Products and Food Branch, Health Canada, Ottawa, ON, Canada; ^3^National Microbiology Laboratory, Public Health Agency of Canada, Winnipeg, MB, Canada

**Keywords:** botulism, genome evolution, horizontal gene transfer, CRISPR

## Abstract

Analysis of more than 150 *Clostridium botulinum* Group II type E genomes identified a small fraction (6%) where neurotoxin-encoding genes were located on plasmids. Seven closely related (134–144 kb) neurotoxigenic plasmids of subtypes E1, E3, and E10 were characterized; all carried genes associated with plasmid mobility via conjugation. Each plasmid contained the same 24-kb neurotoxin cluster cassette (six neurotoxin cluster and six flanking genes) that had split a helicase gene, rather than the more common chromosomal *rarA*. The neurotoxin cluster cassettes had evolved as separate genetic units which had either exited their chromosomal *rarA* locus in a series of parallel events, inserting into the plasmid-borne helicase gene, or vice versa. A single intact version of the helicase gene was discovered on a nonneurotoxigenic form of this plasmid. The observed low frequency for the plasmid location may reflect one or more of the following: 1) Less efficient recombination mechanism for the helicase gene target, 2) lack of suitable target plasmids, and 3) loss of neurotoxigenic plasmids. Type E1 and E10 plasmids possessed a Clustered Regularly Interspaced Short Palindromic Repeats locus with spacers that recognized *C. botulinum* Group II plasmids, but not *C. botulinum* Group I plasmids, demonstrating their long-term separation. *Clostridium botulinum* Group II type E strains also carry nonneurotoxigenic plasmids closely related to *C. botulinum* Group II types B and F plasmids. Here, the absence of neurotoxin cassettes may be because recombination requires both a specific mechanism and specific target sequence, which are rarely found together.

## Introduction

The conventional taxonomic name of *Clostridium botulinum* masks the fact that it is a physiologically diverse group of anaerobic, spore-forming bacteria defined by the ability to produce the deadly botulinum neurotoxin (BoNT), which when ingested by humans or animals can cause the neuroparalytic disease, botulism. Four groups of *C. botulinum* are recognized: Groups I and II primarily cause human botulism, Group III is responsible for botulism in birds and animals, and Group IV has yet to be associated with botulism, although it has been associated with sudden unexpected deaths (Sonnabend 1981; Hatheway 1988; Johnson 2007; Peck 2009). Seven different serotypes of BoNT have been described (A to G). Members of the mesophilic (minimum growth temperature 12 °C) *C. botulinum* Group I possess one to three neurotoxin genes. In some bivalent strains one of these genes may be silent, and in others one gene may be expressed at a much lower rate than the other neurotoxin gene; however up to three different neurotoxins may be formed by the same strain (Dover et al. 2013; Kalb et al. 2014). *Clostridium botulinum* Group I members produce toxin types A, B, and F. *Clostridium botulinum* Group II is genetically and physiologically very different from *C. botulinum* Group I. Strains of *C. botulinum* Group II are psychrotrophs, with a minimum growth temperature of 3.0 °C, and only form one neurotoxin, either type B, E, or F. Until recently members of *C. botulinum* Group II were thought to only possess one neurotoxin gene, but it has recently been shown that strains of *C. botulinum* Group II type F also contain a fragment of a type B and a type E neurotoxin gene, presumably unexpressed (Carter et al. 2013). *Clostridium botulinum* Group III produces neurotoxins of type C and D, or chimeric (C/D or D/C) versions thereof, which are encoded by genes from a bacteriophage genome maintained in the cell as a plasmid (Peck et al. 2011; Skarin et al. 2011). Members of *C. botulinum* Group IV produce neurotoxin type G, and are also known as *Clostridium argentinense*. A Group IV strain from Argentina carries its neurotoxin gene on a 114-kb plasmid (Zhou et al. 1995); however, a recently sequenced strain from Switzerland may have a chromosomal location for its neurotoxin gene cluster (Smith, Hill, Xie, et al. 2015).

Although the presence of plasmids in *C. botulinum* was first noted in the late 1970s and early 1980s (Scott and Duncan 1978; Strom et al. 1984), the first description of human neurotoxigenic *C. botulinum* plasmids was over 20 years later; this was for *C. botulinum* Group I serotypes A and B (Marshall et al. 2007; Smith et al. 2007). Neurotoxigenic *C. botulinum* Group I plasmids vary in size from approximately 149 kb (pCLD of type B1 strain Okra) to 270 kb (pCLJ1 of Ba4 strain CDC 657) (Hill et al. 2009), whereas those of *C. botulinum* Group II type B are much smaller, ranging from 47 to 63 kb (Carter et al. 2014). No synteny has been observed between the neurotoxigenic plasmids of Group I and those of Group II (Hill and Smith 2013; Carter et al. 2014; Carter and Peck 2015). Several *C. botulinum* genomes harbor more than one plasmid; the Group III C/D strain BKT015925 has five, ranging from 12 to 203 kb, the largest of these being the neurotoxigenic phage plasmid p1BKT015925 (Skarin et al. 2011). No *C. botulinum* genomes containing multiple neurotoxigenic plasmids are known; however, there are examples of plasmids bearing two neurotoxin gene clusters; plasmid pCLJ1 of Ba4 strain CDC 657, plasmid pBF of strain Bf, and plasmid pCBG of A2b5 strain CDC 1426 (Marshall et al. 2007; Smith et al. 2007; Hill et al. 2009; Smith, Hill, Xie, et al. 2015). The nonneurotoxigenic *C. botulinum* plasmids often carry genes for fitness or survival, such as bacteriocins (Sebaihia et al. 2007) or other toxins (Skarin et al. 2011).

*Clostridium botulinum* spores persist in the environment in soil and the marine and freshwater sediments of fens, rivers, and lakes. Strains forming type E neurotoxin predominate in sediments of the arctic and subarctic regions (Hielm et al. 1998; Leclair, Farber, et al. 2013), and type E botulism has been noted as a substantial risk to humans in these regions, due to intoxication from poorly preserved food (Dolman et al. 1960; Hauschild and Gauvreau 1985; Austin and Leclair 2011; Fagan et al. 2011; Leclair, Fung, et al. 2013). Twelve subtypes of type E neurotoxin are known, formed by strains that have been isolated from a wide geographical area (Hill and Smith 2013; Hill et al. 2015; Mazuet et al. 2015). Until recently all type E neurotoxin gene clusters were thought to be chromosomally located at the *rarA* gene locus, including those of subtypes E4 and E5, present in neurotoxigenic strains of *Clostridium butyricum* (Macdonald et al. 2011; Smith, Hill, Raphael, et al. 2015). However, as a result of using pulsed field gel electrophoresis (PFGE) to separate intact chromosomes from plasmid DNA species, followed by Southern analysis, it was discovered that three Nordic type E1 strains bore their neurotoxin gene on a plasmid of approximately 146 kb (Zhang et al. 2013). One of these strains, CB11/1-1, was subjected to genome sequencing and the data deposited in the GenBank DNA sequence database, but no plasmid-specific sequences were identified.

In a recent study of Canadian type E strains derived from food, clinical, and environmental sources, data for 175 genomes were obtained by next-generation sequencing (Weedmark et al. 2014, 2015). We present here the results of screening these data for the presence of type E neurotoxigenic plasmids, as well as three newly sequenced Canadian isolates. Together with a type E1 strain from a Hungarian soil sample, and by further analysis of the publically available data for CB11/1-1, we show for the first time that genes encoding subtypes E1, E3, and E10 may all be carried by a family of closely related 133- to 144-kb neurotoxigenic plasmids. Furthermore, annotation of these plasmids has provided evidence for the route by which the more commonly encountered chromosomal type E neurotoxin gene cluster has been derived.

## Materials and Methods

### Culture Conditions, DNA Isolation, and Genome Sequencing

These have been described previously for the Canadian *C. botulinum* type E strains (Weedmark et al. 2014, 2015). *Clostridium botulinum* type E1 strain IFR 12/29 was isolated at the Institute of Food Research (IFR), Norwich, UK, from a soil sample collected at Kiskunmajsa, Bács-Kiskun county, Hungary, using isolation and culture techniques previously described (Peck et al. 2010). Genomic DNA from a 10-ml TYG (tryptone, yeast extract, glucose) broth culture was prepared using standard Gram positive lysis techniques followed by phenol/chloroform purification (Carter et al. 2014). Genome sequencing was performed using the Illumina MiSeq platform, generating 300-bp paired-end reads. After trimming (removal of adaptors and low quality sequence), 15.6 million fastq reads of average length 278 bp were used for contig assembly. Reads were assembled into contigs using SPAdes 3.5.0 software (Bankevich et al. 2012) (http://spades.bioinf.spbau.ru/, last accessed February 26, 2016), using the “careful” setting (enabling error correction and mismatch correction for highest quality assemblies).

### Plasmid Assembly

Plasmids were assembled using CLC Genomics Workbench 8.0.2 (Qiagen). Contigs were extended and further assembled using the Genome Finishing Module 1.5.1 plugin. Initially, contigs containing neurotoxin gene cluster sequences were identified, and 5′ and 3′ ends extended using the original fastq reads until adjoining contigs were reached. Contig junctions were closed by polymerase chain reaction (PCR) amplification and capillary sequencing.

A draft version of plasmid pCB11/1-1 was assembled from publically available whole genome sequence contigs of type E1 strain CB11/1-1 (Zhang et al. 2013; NCBI RefSeq: NZ_AORM00000000.1). Nine plasmid-specific contigs were identified: CB11/1-1 CB_contig0059, 00144, 0081, 0071, 00136, 00156, 0069, 0047, and 0080. Using the map of closely related plasmid p12/29 for comparison, contigs 0059 and 00144 could be joined with no gap; contig 00144 overlapped by 1 bp with contig 0081; this could be joined to contig 0071 with no gap. Between contigs 0071 and 00136 and contigs 00136 and 00156 there was a 9-bp and an 8-bp gap, respectively. Contig 00156 joined to contig 0069 with no gap; between the latter and contig 0047 was a 1-bp gap. The largest gap, of 364 bp, occurred between contig 0047 and contig 0080; this might represent a gene deletion. Contig 0080 overlapped with (starting) contig 0059, although as this overlap region includes an insertion of transposon DNA with respect to p12/29, it was not possible to define the exact junction point for these two contigs. In order to generate a plasmid map for comparative purposes, sequences from p12/29 were used to close contig junctions.

A draft version of the nonneurotoxigenic plasmid from type E1 strain ATCC 17786 (GenBank assembly accession GCA_001276965.1) was similarly assembled by mapping contigs onto the DNA sequence of plasmid p12/29. Plasmid-specific contigs were LHUM01000052 (41,887 bp), LHUM01000009 (39,939 bp), and LHUM01000059 (70,095 bp), which give a minimum plasmid size of nearly 152 kb. Gaps (with respect to p12/29) between contigs were as follows: contigs 52 and 9, 850bp; contigs 9 and 59, an apparent overlap which could not be resolved; and contigs 59 and 52, 106 bp. The chromosomally located (conventional *rarA* locus) type E1 neurotoxin gene cluster was found on contig LHUM01000023 (386,225 bp).

### Annotation

Annotation was performed using CLC Genomics Workbench 8.0.2 to generate predicted peptide gene products of all plasmid open reading frames (ORFs) over 100 codons long with ATG, TTG, or GTG start codons. Each peptide sequence was used as a query to perform a BLASTP (Altschul et al. 1990) or DELTA BLAST (Boratyn et al. 2012) search at NCBI, using default search parameters. DNA sequence comparisons of plasmid and chromosomal DNA were carried out using the Artemis Comparison Tool (ACT) program (https://www.sanger.ac.uk/resources/software/act, last accessed February 26, 2016) (Carver et al. 2005). CRISPR (Clustered Regularly Interspaced Short Palindromic Repeats) spacers and repeats were identified using the CRISPRfinder web service (http://crispr.u-psud.fr/Server/, last accessed February 26, 2016) (Grissa et al. 2007).

### Screen for Nonneurotoxigenic Plasmids

A database of draft *C. botulinum* type E genome sequences of the strains listed in table 1 was generated using CLC Genomics Workbench 8.0.2. A BLASTN query of this database was performed using known plasmid sequences to identify nonneurotoxigenic plasmids.

**Table 1:** *Clostridium botulinum* Type E Strains Used in this Study

Subtype	NP	Isolate				
E1	+	CB11/1-1*	IFR 12-29*			
E1	−	E-RUSS*	E1 Dolman			
E3	+	INGR16-02E1	ST0210E1			
E3	−	211 VH Dolman	FWSK02-04E1	GA9811E2MS	RSKR-68E3	SOKR-43E2
		BE9708E1	FWSK02-05E1	Gordon	S9510E	SOKR-44E3
		CA9708E1	FWSK02-05E2	IG0201E2BC	SE9908E	SOKR-46E3
		F9508EPB	FWSK02-06E1	IG0202E1	SO321E1	SOKR-50E1
		FE0005EJT	FWSK02-06E2	IG0410E2LC	SO325E1	SOKR-50E2
		FE0201E1BC	FWSK02-07E1	IG0410E3LC	SO326E1	SP457-458E8
		FE0202E1TC	FWSK02-07E3	IN01SE63E1	SOKR-18E1	SW279E
		FE0801E1IT	FWSKR4802E1	INWB2202E1	SOKR-19E1	SWKR0402E1
		FE9507EEA	GA0108EJC	ME0702E1CS	SOKR-20E1	SWKR0402E2
		FE9604ENT	GA0202E1TS	ME1010E1JL	SOKR-22E1	SWKR07E1
		FE9708E1JI	GA0702E1	MI9507E	SOKR-22E3	TRK02-02E1
		FE9708E1PI	GA0702E1CS	MI9608ESM	SOKR-23E1	TRK02-02E2
		FE9709EBB	GA0808EPA	MI9706E	SOKR-23E3	TRK02-04E3
		FE9709ELB	GA0811E1IT	MSKR5102E2	SOKR-24E2	TRK02-06E2
		FE9908EDL	GA9604EAK	MU0005EJT	SOKR-24E3	TRK02-06E3
		FE9909ERG	GA9604ESM	MU0103EMS	SOKR-25E2	TRK02-07E1
		FE1010E1JL	GA9608EPB	MU8903E	SOKR-25E3	V9804E
		FWKR02E1	GA9709EHS	MU9708EJG-F235	SOKR-27E1	VI9508E
		FWSK02-01E2	GA9709EJA	MU9708EJG-F236	SOKR-3602E1	VI9608EPB
		FWSK02-01E3	GA9709ENS	RSKR-68E1	SOKR-38E2	VO0202E1TC
		FWSK02-02E1				
E10	+	FI1111E1	FWSKR40E1	SWKR38E1	SWKR38E2	
E10	−	FE9709EBB2	MI69709E	SO303E5	SO309E2	SOKR-49E2
		FWKR11E1	PBKR-41E1	SO304E1	SOKR-33E1	SP417E-Alc
		GA1101E1BB	RSKR-68E2	SO304E2	SOKR-34E2	SP417E-NT
		GA9706EMA	SO303E1	SO305E1	SOKR-34E5	TRK02-04E1
		MI19709E	SO303E3	SO305E2	SOKR-42E1	TRK02-08E1
		MI59709E	SO303E4	SO307E1	SOKR-49E1	TRK02-08E3
E11	−	SO329E1	SOKR-44E1	SOKR-46E1	SW280E	SWKR24E1
		SO329E2	SOKR-44E2			
NT		BE0211E1	BE0211E3	BFLY-2	BFLY-6	FM1101E1BB
		BE0211E2	BFLY-1			

Note.—Strains designated “NT” in “Subtype” column lack a type E neurotoxin gene and carry an intact chromosomal *rarA* gene. The three strains marked with an asterisk derive from Europe; the remainder are from Canada, many of which have been described previously (Weedmark et al. 2014, 2015). NP, neurotoxigenic plasmid present (+) or absent (−).

### Phylogenetic Tree Construction

DNA coding sequences were imported into CLC Genomics Workbench 8.0.2 and used to create sequence alignments. These were used to create unrooted neighbor-joining trees using the Jukes–Cantor nucleotide distance measure, with 100 bootstrap replicates.

## Results

### Neurotoxin Gene Cluster–Bearing Plasmids Discovered in *Clostridium botulinum* Group II Types E1, E3, and E10

Plasmids bearing the type E neurotoxin gene cluster were identified via two routes. Two type E1 strains (IFR 12/29, CB11/1-1) were analyzed following separate PFGE screens. Strain CB11/1-1 (isolated from Finnish whitefish roe) was included following the work of Zhang et al. (2013), and strain IFR 12/29 (isolated from a Hungarian soil sample) was sequenced following a PFGE screen of five type E strains (NCTC 8266, IFR strains 12/23, 12/29, 12/33, 13/03) for plasmid-sized bands hybridizing to a type E neurotoxin gene probe (data not shown). Strain IFR 12/29 possessed an episomal DNA species which migrated at a similar size (approximately 140 kb) to that reported for a PFGE band hybridizing to a type E gene probe from strain CB11/1-1 (Zhang et al. 2013).

Further neurotoxin gene cluster–bearing plasmids were identified following genome analysis. The majority (103) of genomes studied were of type E3, together with 34 type E10, 7 type E11, and 2 type E1 (table 1). Seven additional genomes lacked any detectable neurotoxin gene sequence (data not shown). Six of these strains (BFLY-1, BFLY-2, BFLY-6, BE0211E1, BE0211E2, and BE0211E3) were reported as nontoxigenic in a separate study (Weedmark et al. 2015). BFLY-1, BFLY-2, and BFLY-6 derive from an avian botulism outbreak but were not confirmed as toxigenic, whereas BE0211E1, BE0211E2, and BE0211E3, which are likely to be clonal isolates, as they were from the same sample of beluga whale fat, originally tested positive for type E neurotoxin, but lost toxicity after regrowth of stocks frozen at −80 °C. The remainder of the genome sequence contigs was screened for the presence of a neurotoxigenic plasmid by first searching for an intact version of the chromosomal *rarA* gene. The rationale behind this approach was based on the observation that all known chromosomal versions of the type E neurotoxin gene cluster are located between two halves of a transposon-associated *rarA* resolvase gene that has been split apart by insertion of the neurotoxin gene cluster (Macdonald et al. 2011; Hill and Smith 2013; Mazuet et al. 2015; Hill et al. 2015).This resulted in the discovery of eight genomes containing intact chromosomal *rarA* genes (table 1). Excluding strains which had been isolated from the same outbreak, and any with poor sequence quality, five were chosen for further study: two strains forming type E3 neurotoxin and three strains forming type E10 neurotoxin. All other toxigenic strains, including seven type E11 and both type E1 strains, possessed a neurotoxin cluster at the chromosomal split *rarA* locus.

The complete DNA sequence for each neurotoxigenic plasmid was determined for the following six type E strains: IFR 12/29, INGR16-02E1, ST0210E1, FWSKR40E1, SWKR38E2, and FI1111E1. Assembly of the type E1 plasmid, p12/29, allowed identification of plasmid-specific contigs from the publically available genome sequence data of CB11/1-1, the plasmid of which proved to be closely related to that of strain IFR 12/29. This enabled construction of a draft version of pCB11/1-1. The size of the completed plasmids was found to be 134–144 kb (table 2).

**Table 2:** Type E Neurotoxigenic Plasmid Details and Analysis of CRISPR Spacers

Isolate	Source	Subtype	Plasmid Size (bp)	PCS	PCS Chromosomal Hits (%)	PCS Plasmid Hits (%)	CCS	CCS Chromosomal Hits (%)	CCS Plasmid Hits (%)
IFR12/29	Soil, Kiskunmajsa, Hungary (2012)	E1	138,937	18	0 (0)	7 (39)	89	9 (10)	15 (17)
CB11/1-1	Whitefish roe, Finland (1999)	E1	142,451	15	0 (0)	4 (27)	102	14 (14)	8 (8)
INGR16-02E1	Seal intestine, Kangiqsujuaq, QC, Canada (2002)	E3	134,110	0	0 (0)	0 (0)	12	3 (25)	0 (0)
ST0210E1	Sturgeon, Lake Erie, Canada (2010)	E3	134,110	0	0 (0)	0 (0)	14	3 (21)	0 (0)
FWSKR40E1	Freshwater sediment, Koksoak River, Kuujjuaq, QC, Canada, (2002)	E10	141,186	6	0 (0)	1 (17)	40	11 (28)	2 (5)
SWKR38E2	Seawater, Koksoak River, Kuujjuaq, QC, Canada, (2004)	E10	142,322	6	0 (0)	1 (17)	51	14 (28)	2 (4)
FI1111E1	Atlantic saury, Newfoundland coast, Canada (2011)	E10	143,823	28	0 (0)	3 (11)	22	10 (46)	1 (5)

Note.—The plasmid-borne CRISPR locus, absent in type E3 neurotoxigenic plasmids, comprises six CRISPR-associated *cas* genes plus an array of spacers and repeats (fig. 2). The size of the plasmid identified in strain CB11/1-1 is an estimate based on comparison with closely related plasmid p12/29. Plasmids from type E3 strains INGR16-02E1 and ST0210E1 are identical. Two sets of CRISPR spacers are characterized; those located in a plasmid array are designated PCS, and those on the chromosome are designated CCS. “Hits” refers to a significant BLAST search result (>90% of spacer length, >90% identity with target sequence) when the spacer was used as a query sequence to interrogate the entire bacterial nucleotide NCBI database. CRISPR spacer “plasmid hits” refers to plasmid-borne or chromosomally located CRISPR spacers which identify other plasmid sequences in the database; similarly “chromosomal hits” refer to CRISPR spacers which identify other bacterial chromosomal DNA. PCS, plasmid CRISPR spacers; CCS, chromosomal CRISPR spacers.

**Fig. 1.—:**
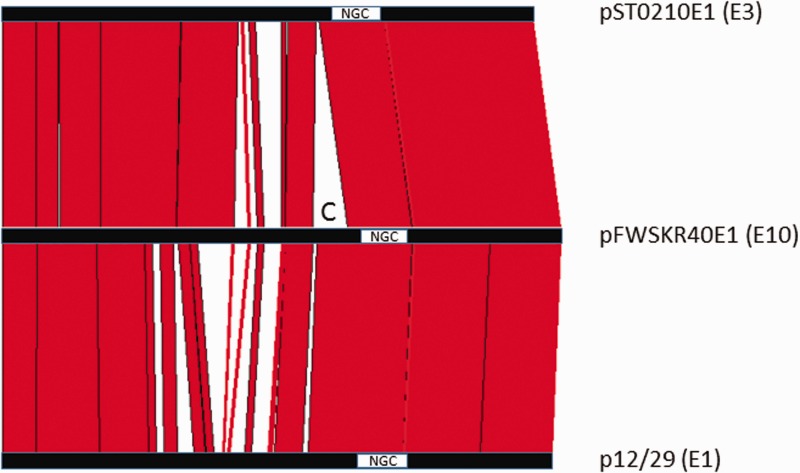
DNA sequence comparison of three classes of neurotoxigenic type E plasmids. The ACT program was used to display blocks of homology (>90% sequence identity) shared between the approximately 140-kb plasmids representing the three classes found in *Clostridium botulinum* Group II type E. The region marked with a C is the CRISPR locus, positioned on the 5′ flank of the neurotoxin gene cluster (see text). This locus is shared between type E1 and type E10 plasmids, but is absent in type E3 plasmids. NGC, neurotoxin gene cluster.

The DNA sequence of representatives of all three plasmid classes (i.e., carrying type E1, E3, or E10 neurotoxin gene clusters) were compared with each other using the ACT program. It was apparent that all plasmids were closely related, with relatively small insertion/deletion differences (fig. 1). The number of raw sequence reads which mapped to either plasmid or chromosomal contigs was approximately equal; this suggests that plasmid copy number may be one per bacterial cell (data not shown).

### Neurotoxigenic Type E Plasmids Include a 24-kb Insert Bearing a Neurotoxin Gene Cluster and an Intact *rarA* Gene, and a Split Helicase Gene

**Fig. 2.—:**
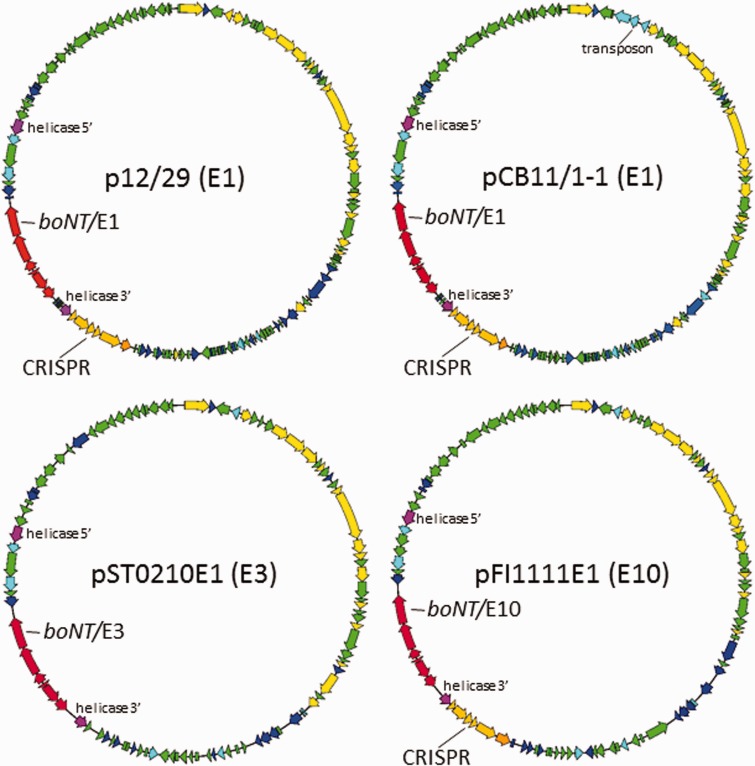
Type E neurotoxin gene cluster bearing plasmids. Representative examples of each main class are shown. **C**oding sequences (CDSs) are colored according to their predicted gene function (see GenBank files for full annotation). Red, type E neurotoxin gene cluster; yellow, plasmid replication/conjugation; green, gene product of unknown function; blue, known gene product not exclusively associated with a plasmid; light blue, bacteriophage-associated gene; light orange, CRISPR-associated *cas* genes; dark orange, CRISPR repeats/spacer array; purple, DEAD/DEAH box helicase gene which has been split by insertion of the 24-kb neurotoxin gene cluster cassette (labeled 5′ and 3′). The three genes colored light blue in pCB11/1-1 indicated by the label “transposon” comprise the main sequence difference between the two type E1 plasmids. Note the lack of CRISPR genes in the type E3 plasmid. Other examples of plasmids carrying the same neurotoxin gene cluster are very similar; plasmid pINGR16-02E1 (E3) is identical to pST0210E1 (E3); plasmid pSWKR38E2 (E10) differs from pFWSKR40E1 (E10) by only a single 1.2-kb gene (a putative Group II intron encoding maturase); plasmid pFI1111E1 (E10) shares 99–100% identity with pFWSKR40E1, differing only in two small (0.9 and 1.4 kb) regions.

Gene organization of representative type E neurotoxigenic plasmids is presented in figure 2. The sequence of the type E3 plasmids from strains INGR16-02E1 and ST0210E1 was identical, although these strains were isolated 8 years apart and from different sources (table 2). The plasmid map for the Finnish type E1 strain CB11/1-1 is very similar to that for the Hungarian strain IFR 12/29 plasmid, apart from a 3.7-kb insertion containing genes for integrases and transposases, which is consistent with a transposition event. Many genes on the type E plasmids encoded predicted products linked to plasmid mobility via conjugation, as observed previously for *C. botulinum* Group II type B4 plasmids (Carter et al. 2014).

**Fig. 3.—:**
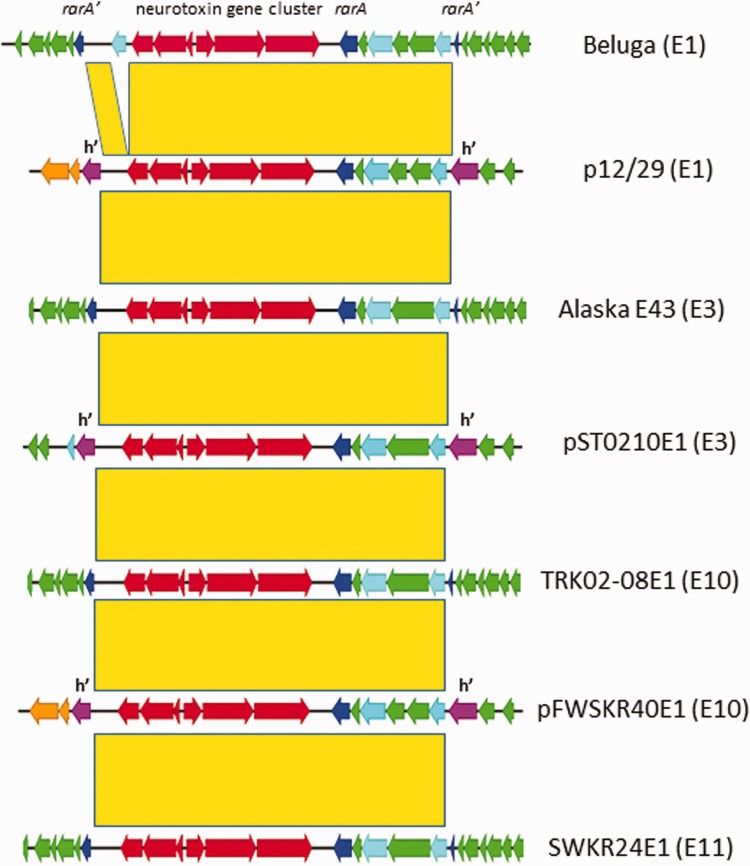
DNA sequence comparison of a 24-kb DNA cassette containing a type E neurotoxin cluster located at a chromosomal or plasmid locus. Redrawn from an ACT comparison; yellow blocks mark regions of high sequence homology (>94% identity). Chromosomal loci for strains TRK02-08E1 and SWKR24E1 were constructed from contigs and raw sequence reads as described for the neurotoxigenic plasmids. CDSs are color coded as in figure 2. The two small, dark blue colored genes marked *rarA*′ are the 5′ and 3′ gene fragments of an original, intact version of *rarA* which has been split by the insertion of the type E neurotoxin gene cluster. These fragments can be seen in all type E strains which harbor chromosomally located neurotoxin gene clusters; note that they are absent from the three plasmid sequences represented here. The larger dark blue genes are the intact versions of *rarA*, located downstream of the *boNT/E* gene. These are present in both chromosomal and plasmid neurotoxin gene cluster loci. Features colored purple and labeled h′ represent the two fragments of a DEAD/DEAH box helicase gene which has been split by insertion of the 24-kb neurotoxin gene cluster cassette. The light blue gene 5′ of the neurotoxin gene cluster in strain Beluga, not shared by the other strains, is predicted to encode a phage integrase/transposase. Note that the two *cas* genes (orange) adjacent to the 3′ half of the split helicase gene are absent from the type E3 plasmid sequence.

Each plasmid contained a conventional type E neurotoxin gene cluster, with (5′ to 3′) *orf-x3*, *orf-x2*, *orf-x1*, *p47*, *ntnh*, and *boNT/E* genes (fig. 3). Comparison with the chromosomally located neurotoxin gene cluster loci showed that a 24-kb piece of DNA was shared between these and the neurotoxigenic type E plasmids. This DNA cassette contained not only the six genes of the neurotoxin cluster, but a further six genes located downstream of the type E neurotoxin gene (fig. 3). Unexpectedly, located on the 3′ flank of each plasmid-borne type E neurotoxin gene cluster was an intact *rarA* gene, as also found in all chromosomal examples of type E neurotoxin gene cluster loci **(**fig. 3**)**. This 24-kb DNA cassette *rarA* gene, encoding a 414 amino acid peptide, follows a different lineage to its split, 420 amino acid encoding counterpart, as reported previously (Hill et al. 2009). On average, the shorter RarA peptide shares 69–70% identity and 79% conserved residues with its longer counterpart. The shorter, 414 amino acid RarA peptide associated with the type E10, plasmid-borne 24-kb cassette is 6 (1.5%) amino acid residues different from the shorter RarA associated with the type E1 and E3 neurotoxin gene clusters, all of which are identical to each other.

The resected, longer *rarA* gene of type E3 strain Alaska E43 is identical to its intact chromosomal counterpart in type E1 strain IFR 12/29; moreover, their gene product differs by only four (0.9%) amino acid residues from the same RarA peptide of the type E3 and type E10 strains which possess an intact, longer rarA gene. This analysis suggests that all of these longer *rarA* genes, whether split or intact, evolved as part of the *C. botulinum* Group II genome, and have not been introduced as a result of horizontal gene transfer.

**Fig. 4.—:**
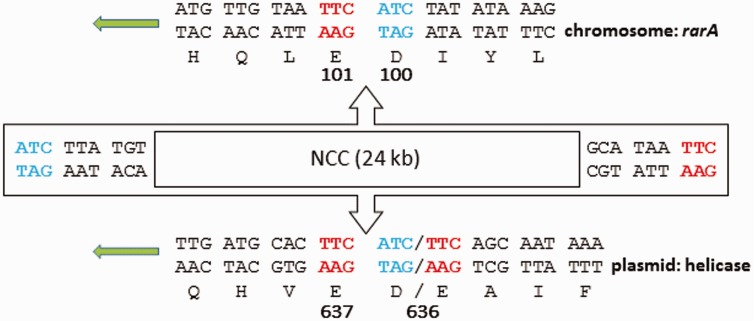
DNA insertion site of type E NCC in the chromosome (*rarA*) or plasmid (helicase). The NCC is positioned with the *orf-x* operon reading from right to left, and the *p47*, *ntnh*, and *boNT/E* operon reading from left to right. Each disrupted CDS reads from right to left, with predicted amino acids designated by their single letter code positioned under the bottom (coding) DNA strand; numbers in bold identify codons flanking the NCC insertion site. DNA sequences in red and blue identify a possible recognition site, 5′-TTCATC-3′, for the recombinase responsible for NCC insertion into the chromosome. This is recreated at each end of the NCC following its insertion into *rarA*, also in the split helicase genes of type E3 and type E10 neurotoxigenic plasmids. In contrast, at each side of the helicase insertion site of type E1 neurotoxigenic plasmids the TTC half of the motif has been repeated. Both versions of codon 636 of the split helicase gene are shown.

The DNA sequence comparison depicted graphically in figure 3 allowed the precise determination of the boundaries between each plasmid and its neurotoxin gene cluster associated (neurotoxin cluster cassette [NCC]) sequence. These boundaries were identical apart from a single base change in the two type E1 plasmids (fig. 4). Once this cassette was deleted from the DNA sequence and the plasmid flanking sequences rejoined, in each case a new ORF was created. This encoded an 1,106 amino acid peptide of the DEAD/DEAH box helicase family (fig. 3), the predicted function of which is the unwinding of nucleic acids. A BLASTP search using this peptide as the query sequence produced only one significant match (99% identity) over its entire length. This match was to a peptide encoded by gene ADT22_16960 from the whole genome sequence of *C. botulinum* Group II type E strain ATCC 17786 (contig 69). A chromosomally located (*rarA* locus) type E1 neurotoxin gene cluster was found on a 386-kb contig from this genome sequence; the entire locus shared 99% identity with that of type E1 strain Beluga. Using the sequence of type E1 plasmid p12/29, it was possible to locate two other plasmid-specific contigs from this genome. Although there were gaps between these contigs with respect to p12/29, these three contigs alone would produce a plasmid of nearly 152 kb. The increased size with respect to the neurotoxigenic type E plasmids can be partly explained by a 17-kb region of DNA which did not match p12/29; this contained several transposase genes. In this respect, it was similar to plasmid pCB11/1-1, which contained a smaller transposon insertion. The presence of these three contigs strongly suggests that strain ATCC 17786 contains a plasmid that is closely related to the neurotoxigenic type E plasmids. This plasmid not only lacks a neurotoxin gene cluster but also carries an intact helicase gene which, in other *C. botulinum* Group II type E strains, has been the target for insertion of a type E NCC. Examination of the insertion site of the 24-kb NCC in the *rarA* and helicase “target” genes showed that a 6 bp motif, 5′-TTCATC-3′, was repeated at each end of the NCC in all split *rarA* genes, and in all type E3 and type E10 split helicase genes. The single base change at the insertion site in the type E1 plasmid helicase had resulted in a duplication of the TTC part of the putative sequence motif (fig. 4).

**Fig. 5.—:**
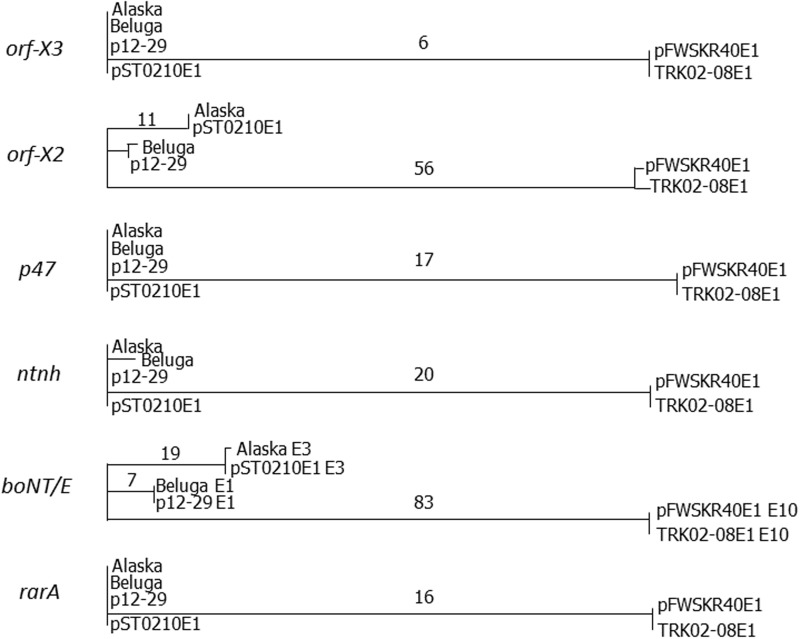
Unrooted neighbor-joining trees of type E1, type E3, and type E10 NCC gene sequences (indicated at the left of each tree). That for gene *orf-x1* is not presented as all examples were identical. Nucleotide differences in basepairs are indicated on major tree branches. An example of a chromosomally located and a plasmid located NCC gene is compared in each tree.

For each type E subtype, plasmid-borne neurotoxin clusters were more closely related (>94% identity) to their *rarA* chromosomal counterparts than to plasmid-borne neurotoxin clusters of other subtypes (data not shown). Individual comparison of plasmid located NCC genes with their chromosomally located counterparts shows that the pattern for the *boNT/E* genes themselves is repeated for the other NCC genes (fig. 5). This was also true for the shorter version of the *rarA* gene associated with the neurotoxin gene cluster (fig. 5).

### Plasmid-Borne CRISPR Loci

On the 5′ flank of the neurotoxin gene cluster in type E1 and E10 plasmids was an approximately 10-kb region comprising a complete CRISPR locus. Adjacent to six *cas* (CRISPR-associated) genes was a region of DNA which contained a typical array of CRISPR repeats and spacers. The CRISPR-*cas* system is a prokaryotic defense mechanism against foreign genetic elements (viruses, transposable elements, and conjugative plasmids) in which short pieces of DNA, termed spacers, copied from these elements are stored in an array, separated from each other by a direct repeat sequence. This locus was entirely absent from the type E3 plasmids (figs. 1–3 and table 2).

**Fig. 6.—:**
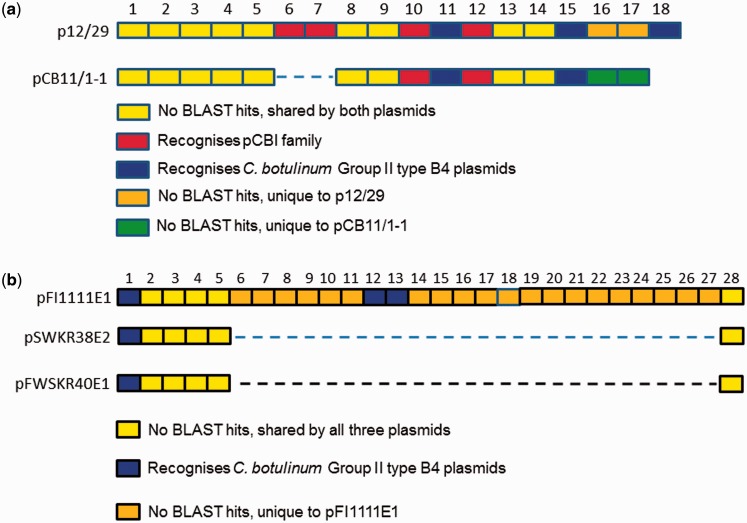
(*a*) Analysis of CRISPR spacers on type E1 plasmids. The pCBI family is named after its first member, discovered in *Clostridium botulinum* Group II type F6 strain Eklund 202F (Smith, Hill, Xie, et al. 2015). Additional family members have been identified in type E strains (this work). (*b*) Analysis of CRISPR spacers on type E10 plasmids.

Plasmid-borne CRISPR spacers, and their chromosomal DNA counterparts, were analyzed for historical evidence of exposure of their bacterial host to invading DNA species (table 2). At each CRISPR locus, spacer length was an average of 35 bp. The organization and content of the plasmid-borne spacer arrays highlighted interesting differences between otherwise closely related plasmids. For the 2 type E1 plasmids, the first 5 spacers were identical, spacers 6 and 7 were missing from pCB11/1-1 with respect to p12/29, and both plasmids shared spacers 8–15. Of most interest, the “newest” spacers differed between the two plasmids (16–18 in p12/29, 16 and 17 in pCB11/1-1, fig. 6a). A similar pattern emerged with the CRISPR spacers of the 3 type E10 plasmids; these also shared the same first 5 spacers; 2 shared spacer number 6, but this same sequence was placed at the end of the spacer array in plasmid pFI1111E1 as a result of the apparent insertion of 22 extra spacers (fig. 6b). The alternative explanation is that 22 spacers have been lost from the middle of the array of pFWSKR40E1 and pSWKR38E2.

BLAST searches using individual spacers as query sequences showed that, of those which returned any significant hits, all recognized plasmids which were related to either the *C. botulinum* Group II type B4 neurotoxigenic family (Carter et al. 2014) or the nonneurotoxigenic plasmid, pCBI, from *C. botulinum* Group II type F6 strain Eklund 202F (Smith, Hill, Xie, et al. 2015). Specifically, of the 28 spacers carried by pFI1111E1, 3 recognized *C. botulinum* Group II plasmids (including spacer number 1, which is shared by the other 2 type E10 plasmids), and of the 18 spacers of p12/29, 9 recognized *C. botulinum* Group II plasmids. All seven *C. botulinum* strains harboring a neurotoxigenic type E plasmid also possessed chromosomally located CRISPR spacers. Analysis of these chromosomal CRISPR spacers revealed that these recognized fewer plasmid sequences, proportionately, compared with those spacers which recognized chromosomal DNA from other *C. botulinum* Group II genomes (table 2), with the caveat that a small proportion of the apparently chromosomal DNA hits were to chromosomally integrated phage genes (data not shown). Interestingly, of all the plasmid or chromosomal spacers analyzed, only one recognized a non–*C. botulinum* Group II plasmid. This was a chromosomal spacer of strain IFR 12/29 which recognized Group III plasmids pC2C203U28, pC2CST, p5BKT015925, pCLG1, and pC2D1873 (Sakaguchi et al. 2009; Skarin et al. 2011). In contrast to those located on the chromosome, plasmid-borne CRISPR spacers did not recognize chromosomal DNA. Significantly, no spacers, whether chromosomal or plasmid borne, identified any known *C. botulinum* Group I plasmids.

### Nonneurotoxigenic Plasmids of *Clostridium botulinum* Group II Type E Are Closely Related to Plasmids of *Clostridium botulinum* Group II Type B and Type F

Having discovered neurotoxigenic type E plasmids and a single example of a closely related nonneurotoxigenic version, the same genome sequence data detailed in table 1 were searched for other plasmid-related contigs. Table 3 summarizes the results, and shows that 11 strains possessed DNA sequence related to 47- to 63-kb plasmids of *C. botulinum* Group II type B4 (Carter et al. 2014), and 31 strains possessed DNA sequence related to pCBI, a nonneurotoxigenic plasmid recently described for *C. botulinum* Group II type F6 strain Eklund 202F (Smith, Hill, Xie, et al. 2015). Four further strains possessed DNA sequences related to both classes of plasmid (table 3). None of these plasmids bore a helicase or *rarA* gene, the known insertion sites of the 24-kb DNA cassette bearing the type E neurotoxin cluster.

**Table 3:** Nonneurotoxigenic Plasmid Sequences Detected in *Clostridium botulinum* Type E Strains

Subtype	Strain	Nonneurotoxigenic Plasmid Type B, F, or B+F	Clade^a^
E3	MI9706E	B	7
E3	SOKR-18E1	B	7
E3	SOKR-24E3	B	7
E3	FE9709EBB	B	16
E3	FE9709ELB	B	16
E3	FE9909ERG	B	16
E3	GA9709EJA	B	16
E3	GA9709ENS	B	16
E3	SO325E1	B	16
E3	SOKR-3602E1	B	16
NT	FM1101E1BB	B	B/F
E3	FWSK02-07E1	F	7
E3	MSKR5102E2	F	7
E3	SOKR-24E2	F	11
E3	FWSK02–04E1	F	22
E3	GA9811E2MS	F	22
E3	MU9708EJG-F235	F	22
E3	MU9708EJG-F236	F	22
E3	SO321E1	F	22
E3	SOKR-20E1	F	22
E3	SOKR-22E1	F	22
E3	SOKR-22E3	F	22
E3	SOKR-23E3	F	22
E3	SOKR-27E1	F	22
E3	TRK02-02E2	F	22
E3	TRK02-04E3	F	22
E10	FI1111E1	F	8*
E10	FE9709EBB2	F	16
E10	GA9706EMA	F	17
E10	RSKR-68E2	F	17
E10	SO303E1	F	17
E10	SO303E3	F	17
E10	SO303E4	F	17
E10	SO304E1	F	17
E10	SO304E2	F	17
E10	SO307E1	F	17
E10	MI59709E	F	21
E10	PBKR-41E1	F	21
E10	SO305E1	F	21
E10	SO305E2	F	21
E10	SOKR-49E2	F	21
E11	SOKR-46E1	F	14
E3	GA0702E1	B+F	22
E3	GA0702E1CS	B+F	22
E3	ME0702E1CS	B+F	22
E10	SP417E-NT	B+F	17

Note.—Sequences detected by BLAST searches in genome sequence contigs are related to *Clostridium botulinum* Group II type B4 plasmids (B), or to *C. botulinum* Group II type F plasmid pCBI (F). Some strains carry both classes (B + F).

a Clade number refers to isolates subjected to phylogenomic profiling by in silico MLST and core SNP analysis (Weedmark et al. 2015; this work). Strain FM1101E1BB is labeled “B/F” in this column as it clusters with *C. botulinum* Group II strains which form either of these two neurotoxin types, together with the type E9 strain CDC 66177 (Weedmark et al. 2015).

*Core SNP analysis of strain FI1111E1 shows that it is distinct from, but most closely related to, clade 8. NT, nontoxigenic.

**Fig. 7.—:**
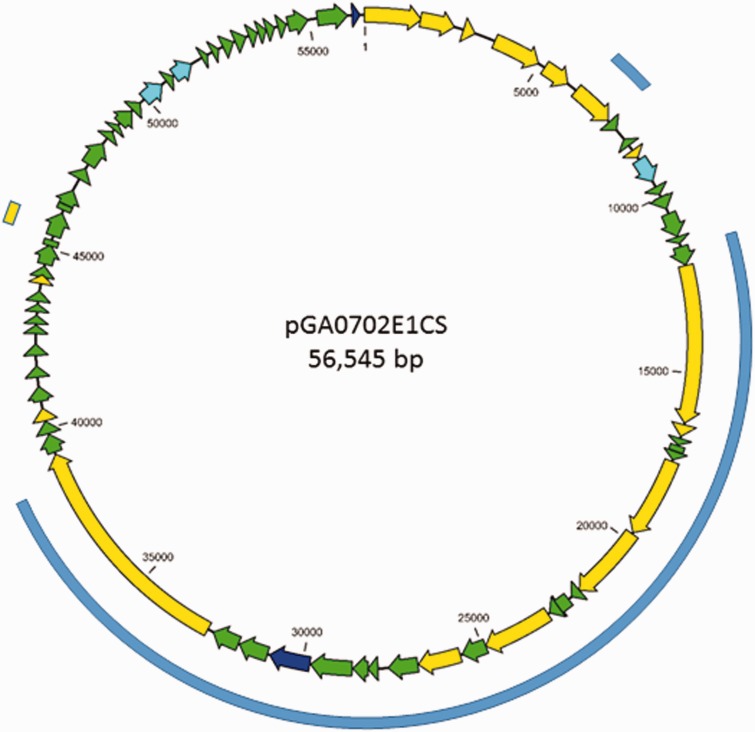
*Clostridium botulinum* Group II type B4 related plasmid family discovered in *C. botulinum* Group II type E strains. CDSs are colored as in figure 2. Outer bars depict regions of homology to other plasmid families; blue, *C. botulinum* Group II type B4 plasmids (87–97% identity); yellow, *C. botulinum* Group II type E1 p12/29 (88% identity). Plasmid pGA0702E1CS only shares a 307-bp (84% identity) sequence with *C. botulinum* Group II type F6 strain Eklund 202F plasmid pCBI. Although most closely related to the neurotoxigenic plasmids of *C. botulinum* Group II type B4, pGA0702E1CS and its close relatives carry no neurotoxin genes.

To demonstrate that these BLAST results indicated the presence of plasmids rather than that of plasmid-related chromosomal sequence, one strain, GA0702E1CS, was chosen for further investigation. Using the same approach as that for identification of the neurotoxigenic type E plasmid contigs, a 56.5-kb plasmid was assembled (fig. 7). An identical plasmid was found in strains SP417E-NT and FM1101E1BB. This plasmid shared at least 50% of its sequence with neurotoxigenic plasmids from *C. botulinum* Group II type B4, with sequence identity ranging between 84% and 99%. A single gene was the only significant source of DNA sequence homology with the neurotoxigenic type E plasmids, the predicted product of which (185 amino acids) shared 90% identity with a hypothetical protein of *C. botulinum* Group II type E1 strain Beluga (encoded by CLO_1413) (fig. 7).

Similarly, three contigs from strain SP417E-NT could be joined to form a 45.5-kb linear DNA molecule which shared 75% of its length with pCBI (40.1 kb) of *C. botulinum* strain Eklund 202F, displaying sequence identity of 87–97%.The SP417E-NT contigs could not be extended to complete this candidate plasmid due to the presence of DNA repeats that were similar in size (105 bp) to the read length for that sequence run.

These results show that nonneurotoxigenic plasmids are relatively common within *C. botulinum* Group II type E; however, those carrying an intact helicase gene which has not yet been split by insertion of an NCC seem to be rare.

## Discussion

The discovery by Lindström and colleagues of neurotoxigenic plasmids in three strains of *C. botulinum* Group II type E1 (Zhang et al. 2013) was important, as previously all studied strains of *C. botulinum* Group II type E and neurotoxigenic *C. butyricum* type E carried their neurotoxin gene cluster at the chromosomal *rarA* gene locus. In this study, neurotoxigenic plasmids have been discovered in another type E1 strain, and also now in strains forming type E3 and E10 neurotoxin, additionally extending their range from Europe to North America. Taking account of data from previous work (Zhang et al. 2013) and this study, approximately 6% (12/194) of all strains studied carried a neurotoxigenic plasmid. Although strains carrying these plasmids were found for subtypes E1 (4/18), E3 (2/109), and E10 (4/34), none were found for subtypes E2, E6, or E11, although altogether only 13 strains from these 3 subtypes were analyzed. We have presented for the first time the complete DNA sequence of all three classes of plasmid, which are closely related and display a limited size range of 134–144 kb, confirming the original PFGE size estimate (Zhang et al. 2013). These type E neurotoxigenic plasmids fall between the size range of the *C. botulinum* Group II type B4 neurotoxigenic plasmids (47–63 kb), and the 149–270 kb neurotoxigenic plasmids of *C. botulinum* Group I (Carter et al. 2014; Marshall et al. 2010). As with other *C. botulinum* Group II neurotoxigenic plasmids, and examples from *C. botulinum* Group I, these possess a set of genes whose predicted products suggest that they may be capable of mobility via conjugation (Marshall et al. 2010; Carter et al. 2014). The discovery of neurotoxigenic plasmids for only 3 of the 12 type E neurotoxin subtypes may be a reflection of the number of genome sequences available for study. Those for types E1, E3, and E10 are the most numerous, and if, based on this study, the proportion of strains with neurotoxigenic plasmids compared with those possessing chromosomally located neurotoxin gene clusters is approximately 6%, then it might be expected that neurotoxigenic plasmids for the other subtypes may be discovered as more strains are characterized. Furthermore, strains with neurotoxigenic plasmids may possess specific genetic traits (in addition to possession of a suitable target plasmid) which predispose them to loss of the NCC from the chromosome. When *C. botulinum* type E genomes were subjected to core single nucleotide polymorphism (SNP) and in silico multi locus sequence typing (MLST) analysis, three type E10 strains now known to contain these plasmids (FWSKR40E1, SWKR38E1, and SWKR38E2) formed a distinct lineage which separated them from other type E10 strains from the same geographical location (Weedmark et al. 2015). In a similar analysis of strains analyzed in this study (data not shown), neurotoxigenic plasmid bearing type E1 strains IFR 12/29 and CB11/1-1 did not cluster together, but were closely associated with type E1 strains possessing a chromosomal neurotoxin gene locus. Type E3 neurotoxigenic plasmid bearing strains INGR16-02E1 and ST0210E1 clustered with type E10 strain FWKR11E1 (clade 4) and type E10 neurotoxigenic plasmid bearing strain FI111E1 clustered with type E3 strain 211 VH Dolman (clade 8). Each of these clades is genetically well separated from its neighbors (Weedmark et al. 2015), suggesting that neurotoxigenic plasmids may be confined to a specific subset of the *C. botulinum* population.

An unexpected and significant discovery was that the same 24-kb DNA cassette was present in both plasmid-borne and chromosomal-borne type E neurotoxin gene cluster loci in *C. botulinum* Group II and neurotoxigenic *C. butyricum*. This cassette included an intact *rarA* gene at the 3′ flank of each type E neurotoxin gene cluster (Hill et al. 2009; Hassan et al. 2014). RarA is a resolvase involved in recombination or insertion events of transposons (Hill et al. 2009). It has been postulated that interaction of the neurotoxin cluster–associated *rarA* gene with its chromosomal counterpart is a key component of the mechanism by which chromosomal integration of the type E neurotoxin gene cluster occurs, splitting the latter into two pieces (Hill et al. 2009).

A copy of the longer, 420 amino acid encoding version of the *rarA* target gene which is split by insertion of the chromosomally located 24-kb DNA cassette is completely absent from the neurotoxigenic plasmids. As stated above, only the shorter version of the *rarA* gene encoding 414 amino acids is found in these plasmids, located in exactly the same position 3′ of the *boNT/E* gene as it is when found on the chromosome. There are at least two possible interpretations for the presence of the same 24-kb DNA cassette in both plasmid-borne and chromosomal-borne loci. One interpretation is that the neurotoxigenic plasmids have received a version of the 24-kb DNA cassette from the chromosome. Alternatively, the neurotoxigenic plasmids are the vehicles by which this cassette is delivered to the *rarA* chromosomal locus. This also does not exclude the possibility that this apparently mobile DNA cassette could be passed from plasmid to plasmid within the same cell. There is no trace of *rarA* gene sequence at the borders of the 24-kb NCC in the type E plasmids, suggesting that if the chromosome to plasmid route has been followed then this must have happened by a very precise recombination mechanism. However, there is precedent for just such a mechanism. In *Bacillus subtilis*, the gene for transcription factor σ^K^ exists in the chromosome as two separate pieces (*spoIVCB* and *spoIIIC*) separated by a 42-kb intervening sequence. This is deleted following a reciprocal recombination event, bringing together the two halves of the ORF to form a functional σ^K^ gene. The rearrangement and deletion of this intervening piece of DNA is controlled by the product of a recombinase gene (*spoIVCA*) that is located on the dispensable 42-kb DNA element (Kunkel et al.1990).

The high proportion of strains bearing a chromosomal NCC compared with those with plasmid versions suggests that any possible equilibrium between chromosome and plasmid favors the chromosome. The discovery that neurotoxigenic plasmids for three different subtypes of the type E neurotoxin gene are so closely related, both in size and gene content, supports the idea that the original integration of the NCC (or its piece by piece assembly) into a plasmid has been a very rare event, and that most or perhaps all of the current neurotoxigenic type E plasmids have arisen through a series of parallel insertions into a single progenitor. The presence in all versions of the type E neurotoxigenic plasmid of a DEAD/DEAH box helicase gene that has been split by insertion of the 24-kb NCC adds strength to this hypothesis. The fact that, in nearly 200 *C. botulinum* type E genomes currently available for study, only one nonneurotoxigenic plasmid was found that carries an undisrupted version of this helicase gene also supports the view that these recombination events are rare. Additionally, low stability of the type E neurotoxigenic plasmid may also contribute to a low frequency of plasmid-borne type E neurotoxin genes, and also the common observation that type E strains may lose their neurotoxin genes. The possibility should also be noted that loss of toxigenicity may not be solely due to loss of a neurotoxigenic plasmid; the NCC could be lost from its chromosomal site but fail to be transferred to a suitable target plasmid. Because the neurotoxigenic type E plasmids are found in strains which were isolated from both Europe (Finland and Hungary) and North America (Canada), this further implies that availability of a suitable target plasmid occurred before these strains became geographically separated. An interesting, but currently unexplainable point is that although type E1 neurotoxigenic plasmids have been found in European but not North American strains, chromosomal type E1 genes have been isolated from both geographical regions. Comparison of the intact version of the plasmid helicase gene from ATCC 17786 with the split versions of the neurotoxigenic plasmids together with nontargeted and resected *rarA* genes enabled the discovery of a putative 6 bp motif, 5′-TTCATC-3′, at each end of the NCC in both split *rarA* genes and in type E3 and type E10 split helicase genes. It is possible that this 6 bp motif may be the recognition site of the RarA enzyme needed for DNA recombination. The observation that this motif is intact at each end of the NCC suggests that the same site may be used for precise excision of the NCC, restoring the function of the original *rarA* gene. The two examples of type E1 neurotoxigenic plasmid each have a single base change in this motif which might prevent loss of the NCC from the plasmid by this mechanism.

Prior to this study, insertion of a *C. botulinum* neurotoxin gene cluster into the coding sequence of a gene was not confined to a single set of *rarA*/type E examples. In all strains (five) of *C. botulinum* Group II type F6 that have been studied, a 34-kb DNA sequence, which includes the type F6 neurotoxin gene cluster, has been inserted into a *topB* gene which encodes DNA topoisomerase III. As with *rarA*, an extra copy of *topB* has accompanied the neurotoxin gene cluster (Carter et al. 2013). It could be argued that circumvention of the normal host defenses against invading foreign DNA may require the disruption of a gene which is involved in DNA recombination, as are *rarA*, *topB*, and now the plasmid-borne helicase. However, a further example may cast doubt on this hypothesis. The type F4 neurotoxin gene cluster in *C. botulinum* Group I strain Af84 has split the *pulE* gene in a similar manner to that seen for *rarA*, *topB*, and the helicase (Dover et al. 2013). The *pulE* gene encodes not a recombinase but an enzyme involved in a secretion pathway that may be used for pilus assembly (Ayers et al. 2010). The fact that the same *pulE* gene has also been split in *C. botulinum* Group I type A3 strain Loch Maree, but in this case the invading DNA contains no neurotoxin gene cluster (Dover et al. 2013), suggests that site preference for DNA recombination, at least in *C. botulinum* Group I, may rely on a recombination hotspot that is found in only a few places in a genome. There is one significant difference between the NCC inserted into the *rarA* and *topB* genes and the one inserted into the plasmid-borne helicase gene. In the former two examples a replacement *rarA* or *topB* gene has been supplied by the invading cassette, presumably because the target gene has an essential function. No such intact helicase gene exists in the type E NCC, whether chromosomal or plasmid borne. This suggests that in contrast to *rarA* and *topB*, the DEAD/DEAH box helicase is not an essential gene.

A CRISPR locus (*cas* genes plus spacer array) can act as a single operational unit that can be moved horizontally, from plasmid to plasmid and between plasmid and chromosome (Horvath and Barrangou 2010). In this respect it mimics the neurotoxin gene cluster, and the fact that it is found in both type E1 and type E10 plasmids directly adjacent to the neurotoxin gene cluster may not be a coincidence; as with the chromosome, where it has been shown that there are hotspots for insertion of the neurotoxin gene cluster (Hill et al. 2009), and as discussed above, there may also be similar regions within these plasmids which accept integration of new DNA species more readily. The fact that the type E3 plasmids lack a CRISPR locus may suggest that these plasmids represent a lineage that has not yet come into contact with the source of this horizontally acquired locus; alternatively, the entire locus may have been moved from the neurotoxigenic plasmid to another plasmid or chromosome. A CRISPR locus was also discovered in some of the 610–825 kb megaplasmids of Italian strains of neurotoxigenic *C. butyricum* type E. This locus was part of a horizontally transferable 168-kb region which was absent from the smaller megaplasmids (Iacobino et al. 2013). All neurotoxigenic strains of *C. butyricum* so far described, however, contain the chromosomal version of either the type E4 or E5 neurotoxin gene locus, hence these megaplasmids are not *C. butyricum* counterparts of the *C. botulinum* neurotoxigenic plasmids described in this study.

When both chromosomal and plasmid-based CRISPR spacers are examined, there is an apparent difference between the two European type E1 CRISPR spacers and those of types E3 and E10 in terms of evidence of exposure to invading plasmid DNA. However, the fact that type E strains with neurotoxigenic plasmids are less commonly isolated than those with chromosomal loci means that the low numbers available for examination make it difficult to draw any definite conclusions in this respect. It is also not known how many of the CRISPR spacers that failed to give a positive BLAST search hit might recognize plasmid sequences that have not yet been described.

Although the two type E1 plasmids are obviously closely related, and both come from Europe (Finland and Hungary), there are two interesting differences. Apart from an extra region containing three integrase genes in pCB11/1-1, probably the result of a transposon insertion, the spacers of the CRISPR locus may provide evidence of the point in evolution where the two strains diverged/became geographically separated, as, ignoring the two missing/inserted spacers in the center of the array, the last few spacers (i.e., at the newest end) differ from each other. More study of *C. botulinum* CRISPR systems would be needed to add weight to this hypothesis, however, as it is known that conservation of the spacer profile varies widely with bacterial species; those of *Streptococcus thermophilus*, for instance, display an extensive diversity, whereas those of *Escherichia coli* are much more conserved (Touchon et al. 2011; Lopez-Sanchez et al. 2012).

One of the most interesting observations was that, despite performing BLAST searches of each spacer sequence against the entire bacterial nucleotide database, significant hits were almost exclusively confined to plasmids of *C. botulinum* Group II. At first, this seemed to provide evidence for why *C. botulinum* Group II strains are not found with more than one complete neurotoxin gene: the immunity to invasive DNA provided by a functional CRISPR system. However, a further screen of all the type E genomes revealed that the CRISPR spacers are not necessarily defending against “foreign” plasmids from *C. botulinum* Group II types B and F; very close relatives of these plasmids, both neurotoxigenic and nonneurotoxigenic, are prevalent throughout the type E population. In this respect, the CRISPRs of *C. botulinum* Group II type E seem to mimic those of *Streptococcus agalactiae*, in which more than 40% of its CRISPR spacers identified mobile genetic elements found in *S. agalactiae* genomes. This suggests that rather than acting exclusively as a defense system, the *S. agalactiae* CRISPR system is modulating the diversity of mobile genetic elements in the overall bacterial population (Lopez-Sanchez et al. 2012).

As mentioned above, there are examples of *C. botulinum* Group I and Group II plasmids which not only have genes for mobility via conjugation, but which have been proven experimentally to perform this function (Marshall et al. 2010). As such, the observation that none of the *C. botulinum* Group II type E CRISPR spacers identify Group I plasmids was surprising. Together with the clear difference between the evolutionary dynamics of horizontal transfer of neurotoxin gene clusters (Carter et al. 2009; Stringer et al. 2013; Carter and Peck 2015), this adds to the evidence that these two groups are genetically, if not geographically, isolated from each other.

In summary, comparison of the type E neurotoxigenic plasmid sequences with their chromosomal counterparts has enabled the precise definition of the NCC that has been inserted into the original plasmid. This cassette is 24-kb long, and contains the six neurotoxin cluster genes and an additional set of six genes located downstream of the type E neurotoxin gene. At present, neurotoxigenic plasmids are only known for types E1, E3, and E10. Because these three subtypes are also the most commonly sequenced examples, it is possible that neurotoxigenic plasmids for other type E subtypes will be discovered as more strains are isolated and their genomes sequenced. Phylogenetic analysis suggests that the NCC has evolved as a separate genetic unit; each set of six neurotoxin cluster genes (and the associated *rarA* gene) appears to have remained together as each type E subtype has evolved. At some point following this divergence, we speculate that either some NCCs have exited their *rarA* locus and have reinserted into a new target gene, a DEAD/DEAH box helicase on a plasmid, in a series of parallel events, or conversely that the neurotoxigenic plasmids have acted as vehicles for delivery of the NCC into the *rarA* gene in the chromosome. One example is known of a nonneurotoxigenic version of this plasmid in a *C. botulinum* type E background; this may represent a plasmid which has already delivered its NCC, or one which has yet to receive an NCC from the chromosome. The apparent ratio of type E strains with chromosomal neurotoxin gene clusters to those with neurotoxigenic plasmids may reflect the rarity of chromosome to plasmid NCC transposition, which could be due either to a less efficient recombination mechanism for the helicase target when compared with the *rarA* target, or to a lack of suitable target plasmids in the bacterial cell. However, this ratio may also simply be the result of neurotoxigenic plasmid loss, or excision of the NCC from its *rarA* locus and failure to reintegrate into a suitable plasmid (in either case strains would not readily come to our attention). Finally, families of plasmids have been discovered which exist in the *C. botulinum* type E population that have close sequence similarity to those known from *C. botulinum* Group II type B and type F. The fact that none of these have become targets for insertion of an NCC supports the hypothesis that this form of recombination requires a specific mechanism, possibly combined with a specific target sequence, both of which are found only rarely.
